# Mineral and Organic Fertilizers’ Effect on the Growth of Young Argane Trees (*Argania spinosa* L.) and Soil Properties under Vulnerable Conditions

**DOI:** 10.3390/plants13152026

**Published:** 2024-07-24

**Authors:** Naima Chabbi, Said Labbassi, Chaima Afi, Salahddine Chafiki, Maryem Telmoudi, Fatima Ezzahra Tiouidji, Ahmed Wifaya, Rachid Bouharroud, Abdelghani Tahiri, Redouan Qessaoui, Khadija Bendiab, Driss Hsissou, Naima Ait Aabd, Abdelaziz Mimouni

**Affiliations:** 1 Regional Center of Agricultural Research of Agadir, National Institute of Agricultural Research (INRA), Avenue Ennasr, BP415 Rabat Principale, Rabat 10090, Morocco; 2 Laboratory of Agrobiotechnology and Bioengineering, Department of Biology, Faculty of Science and Technology-Gueliz, Cadi Ayyad University, Marrakesh 40000, Morocco; 3 Laboratory of Biotechnology and Valorization of Natural Resources (LBVRN), Faculty of Sciences, Ibn Zohr University, Agadir 80000, Morocco; 4 AgroBioSciences Department, Mohammed VI Polytechnic University, Lot 660, Hay Moulay Rachid, Ben Guerir 43150, Morocco; 5 Laboratory of Environmental, Ecological and Agro-Industrial Engineering (LGEEAI), Faculty of Science and Technology of Beni Mellal, Sultane Molay Slimane University, Beni Mellal 23000, Morocco

**Keywords:** *Argania spinosa*, compost, NPK, frequency, plant physiology, soil parameters

## Abstract

*Argania spinosa* (L.) Skeels is an endemic species to Morocco that has multiple uses. It plays multiple important roles in terms of its botanical, ecological, and economic properties. However, the domestication of this species will open up considerable economic opportunities for Morocco. Here, for the first time, we assessed the effect of different doses of compost and NPK fertilizers on the vegetative growth parameters, biochemical and antioxidant potential of the *Argania spinosa* plant, and soil properties. Over a two-year period (2022–2023), eight different treatments were applied across two experimental sites. These treatments included the following: T0 (Control), T1 (F1-80.50.70 g NPK/plant), T2 (F1-125.75.100 g NPK/plant), T3 (F2-160.100.140 g NPK/plant), T4 (F2-250.150.200 g NPK/plant), T5 (F1-2.5 kg/plant compost), T6 (F1-5 kg/plant compost), T7 (F2-5 kg/plant compost), and T8 (F2-10 kg/plant compost), with F1 and F2 being the frequencies of application. We compared several doses of fertilizers with no fertilization as a control. The results showed a significant influence of the compost and NPK fertilizer on the vegetative growth parameters. For the Tamjlojt site, the first year is important because treatments T3 and T4 significantly increased height by 71.94 ± 21.15% and 74.31 ± 12.31%, respectively. For the circumference, the results showed a significant improvement by the treatments T4 and T3, and T1 demonstrated the highest gain. For the collar diameter, all treatments showed a significant difference. The most notable difference was observed with treatments T3 and T7 with 115.63 ± 33.88% and 101.09 ± 20.84%, respectively. For the Rasmouka site, the second year was the most important. The treatments with the most important height increase were T7 and T8, with a value of 43.14 ± 10.06% and 36.44 ± 9.95%; the same was observed for collar diameter as a significant increase was found in T8 and T7 with a value of 55.05 ± 15.7% and 54.08 ± 9.64%. For the circumference parameter, the treatments that increased significantly this parameter were T8 and T7 with a value of 53.36 ± 15.11% and 50.34 ± 11.29% in 2023. In addition, the highest content of carbohydrates was recorded for the treatment T3 with a value of 148.89 ± 8.11 (mg EG/g). For phenolic determination, the highest value was 2532 ± 457.13 (µg GAE/mL), shown for treatment T1. For flavonoids, the treatments that showed a significant effect were T1 and T6 with a value of 2261.98 ± 184.61 and 1237.70 ± 95.65 (µg QE/mL), respectively. For the impact on soil properties, the electrical conductivity, at the Tamjlojt site, treatment T1 showed a significant increase to 1139.00 ± 241.30 (ms/cm), while at the Rasmouka site, treatment T8 showed a significant increase to 303.33 ± 9.33 (ms/cm). Concerning organic carbon, all treatments resulted in increased percentages of this parameter in the soil. For the Tamjlojt site, the T7 treatment had a significant positive effect on this parameter with a value of 0.87 ± 0.12%. For the Rasmouka site, the T3 treatment increased the percentage of organic carbon with a value of 1.17 ± 0.07%. In addition, the organic matter content showed an improvement with a value of 2.02 ± 0.12%. As there are no previous studies in *Argania spinosa* fertilization, this study greatly contributes to our understanding of the benefits of using different fertilizers at different doses, in particular T8 and T7 as organic fertilizers and T3, T4 as chemical ones, on argan growth, the biochemical and antioxidant properties of leaves, and its soil properties.

## 1. Introduction

Mineral and organic fertilizers play an extremely important role in the enrichment of soil fertility, which, in turn, promotes plant growth [1]. Inorganic and organic fertilizers have an important role in increasing agricultural production [2]. Presently, the most commonly used fertilizers are NPK fertilizers, and chemical fertilizers might be essential because they can re-establish soil fertility very quickly and the nutrients become available to the plants as soon as the fertilizers are dissolved [3]. However, these can have negative consequences on ecosystems, such as soil degradation, which can occur through physical factors like structural decline, crusting, compaction, erosion, anaerobic conditions, and water imbalance. Chemical factors include acidification, salinization, elemental imbalances leading to toxicity or deficiency, and nutrient depletion. Biological degradation involves the depletion of soil organic carbon pools, a reduction in soil biodiversity, and a decline in microbial biomass carbon [4]. Additionally, chemical fertilizers can contribute to groundwater pollution, surface water eutrophication, and emissions of greenhouse gases [5,6,7]. Organic fertilizers (manure or crop residues) are effective in promoting environmental sustainability and plant growth after long-term use [8]. Macronutrients are important nutrients for plant growth. Nitrogen, phosphorus and potassium are the prime macronutrients that help plant growth and development [9,10]. It is well-known that nitrogen (N) is a limiting factor in crop production and that fertilization is necessary for vegetative growth (leaves, stem, and roots) and a high yield of good quality [11]. Moreover, it is also the main component of proteins, amino acids, nucleic acids, chlorophyll, and phytohormones, and therefore it plays a major role in plant metabolic processes [12,13]. Moreover, phosphorus (P) is the second limiting macronutrient that plays a pivotal role in regulating plant energy generation through ATP synthesis and various transport mechanisms [14]. And it can enhance resistance to environmental stresses while improving overall growth and development as well as increasing crop productivity [15,16]. In the same way, potassium (K) is one of the most important plant nutrients and is essential for plant growth, metabolism, and development [17]. It is a highly mobile element and is uptaken by plants as a K^+^ ion. It is the most abundant cation in living plant cells and plays an important role in osmotic adjustment, protein synthesis, membrane polarization control, carbohydrate metabolism, and enzyme activation [18,19].

*Argania spinosa* (L.), an endemic tree of center-western Morocco, belongs to the Sapotaceae family [20]. It plays an important ecological, environmental and socio-economic role. Firstly, it is a tree found in arid areas, protecting the soil from erosion and slowing down the drying process while shading other crops and maintaining soil fertility [21,22]. Secondly, the wood is used as fuel and the leaves as ‘hanging forage’ to feed goats and other animals [23,24]. Thirdly, it is a very important source of economic income for the people who exploit it, mainly for its oil importance [23]. Despite its importance, the argan tree population is decreasing drastically in both density and area, and its natural regeneration is practically absent [25,26]. However, water deficiency and soil infertility considerably reduce the success of plantations [27]. For *Argania spinosa,* which grows naturally in semi-arid conditions and has adapted to harsh environments, understanding the effects of nutrients on its growth and development is essential to its eventual domestication. Nutrients such as nitrogen, phosphorus and potassium play a fundamental role in the physiological processes of plants, influencing everything from root development to flowering and fruiting. Studying the interaction of these nutrients with *Argania spinosa* could provide valuable information on optimizing its growth, increasing fruit production and, ultimately, facilitating its adaptation to cultivated environments.

The scientific hypothesis was proposed suggesting that fertilizing *Argania spinosa* with essential nutrients such as nitrogen, phosphorus, and potassium could potentially enhance its growth and reproductive success, increasing growth rates and enhancing resistance to environmental stresses.

The purpose of this research was to evaluate the impact of different doses of compost as organic fertilizers [28] and NPK as a chemical one under agro-climatic conditions of two different sites in Morocco on improving the growth characteristics, soil proprieties and biochemical and antioxidant potential of *Argania spinosa*.

## 2. Results

### 2.1. Vegetative Growth Parameters

**Figure 1:**
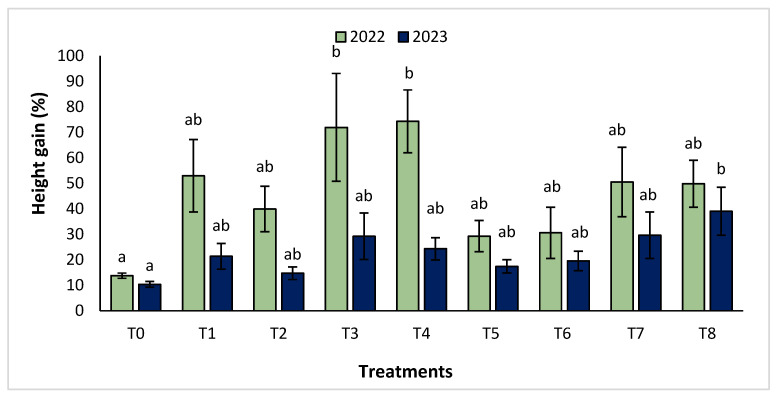
Height gain (%) for the Tamjlojt site in two years (2022–2023). The bars with the same letters are not significantly different at a 5% significance level, according to the Tukey test. Error bars refer to standard errors.

**Figure 2:**
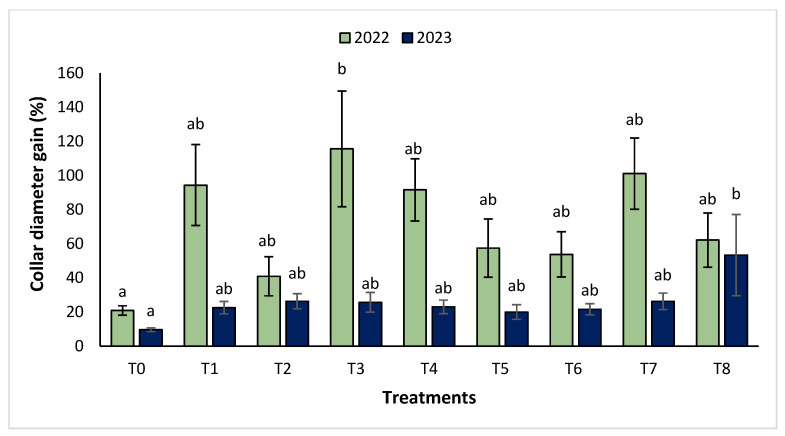
Collar diameter gain (%) for the Tamjlojt site in two years (2022–2023). The bars with the same letters are not significantly different at a 5% significance level, according to the Tukey test. Error bars refer to standard error.

**Figure 3:**
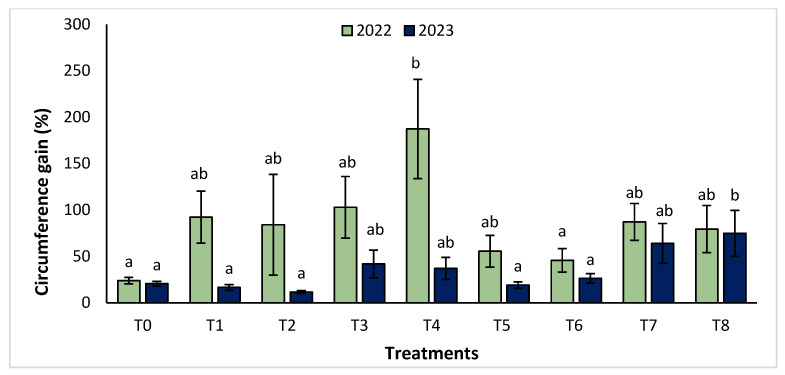
Circumference gain (%) for the Tamjlojt site in two years (2022–2023). The bars with the same letters are not significantly different at a 5% significance level, according to the Tukey test. Error bars refer to standard errors.

The agronomic performance of *Argania spinosa* in the two studied sites was significantly influenced by inorganic fertilizer (NPK) and organic (compost) treatments compared to the control. In the first year, for the Tamjlojt site, all organic and inorganic treatments improved agronomic parameters (height rate, collar diameter, and circumference) (Figure 1, Figure 2 and Figure 3). For height rate, the results showed that treatments T3 and T4 significantly increased height with percentages of 71.94 ± 21.15% and 74.31 ± 12.31%, respectively, followed by T1, T7 and T8 with percentages of 52.95 ± 14.19%, 50.52 ± 13.62% and 49.79 ± 9.22%, respectively. Thus, for the collar diameter, all treatments showed a significant difference. The most notable difference was observed with treatments T3 and T7 with 115.63 ± 33.88% and 101.09 ± 20.84%, respectively. The least significant difference was observed with T2, with a gain of 40.98 ± 11.45% compared to the control, which was 20.92 ± 2.77%. Hence, for the circumference, the results showed a significant improvement with the treatments. The treatments T4, T3, and T1 demonstrated the highest gain. The lowest gain was in T6 with 45.67 ± 12.64%, which did not exhibit any significant difference when compared to the control’s gain of 23.96 ± 3.56%.

In the second year, at the same site, both organic and inorganic treatments increased the measured agronomic parameters. For height rate, the results showed that the treatments T8, T7, T3, and T4 improved the gain with the following percentages 39.02 ± 9.44%, 29.65 ± 9.10%, 29.24 ± 9.11% and 24.31 ± 4.35%, respectively, compared to the control.

The results revealed that the treatments were effective in increasing the collar diameter. The treatment that most significantly increased this parameter was T8 (53.35 ± 23.76%), followed by T7 (26.30 ± 4.80%), and T2 with a gain of 26.31 ± 4.43% compared to the control. However, the smallest increase was observed with T5 with a diameter of 20.02 ± 4.27%. For the measurement of circumference, the treatments showed a significant difference, with T8 followed by T7, T3, and T4 with values of 74.86 ± 24.72%, 63.99 ± 21.37%, 41.86 ± 14.96% and 37.10 ± 11.75%, respectively. The other treatments (T1, T2, T5, and T6) showed no significant difference compared to the control.

**Figure 4:**
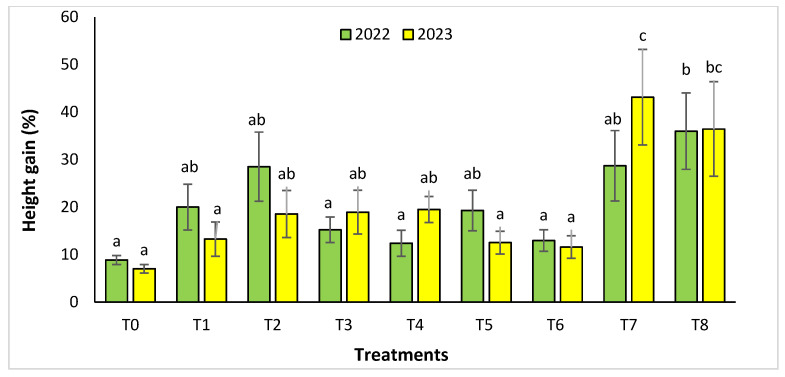
Height gain (%) for the Rasmouka site in two years (2022–2023). The bars with the same letters are not significantly different at a 5% significance level, according to the Tukey test. Error bars refer to standard errors.

**Figure 5:**
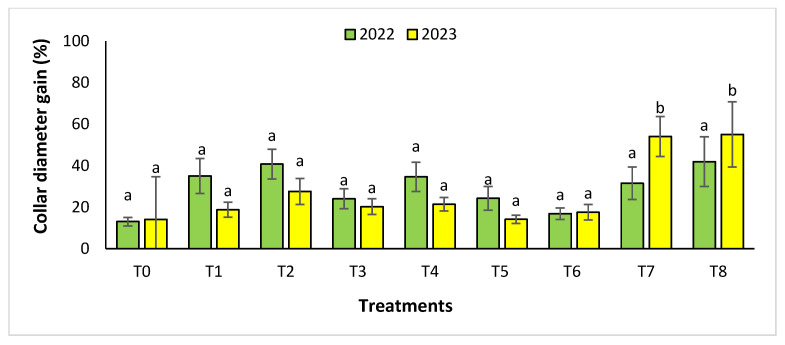
Collar diameter gain (%) for the Rasmouka site in two years (2022–2023). The bars with the same letters are not significantly different at a 5% significance level, according to the Tukey test. Error bars refer to standard errors.

For the Rasmouka site, the height gain increased significantly in the two years (Figure 4). In 2022, the highest gain was observed in the treatments T8, T7, and T2 with a value of 35.99% ± 8.04, 28.69 ± 7.41% and 28.51 ± 7.29%, respectively, followed by T1, T5, with a gain of 20.01 ± 4.81% and 19.3 ± 4.26%. In 2023, for the same parameter, the treatments with an important increase were T7 and T8 with a value of 43.14 ± 10.06% and 36.44± 9.95%, followed by T4, T3, and T2 with a gain of 19.5 ±2.76%, 18.95 ± 4.61% and 18.57 ± 4.96%, respectively. For collar diameter (Figure 5), in the second year, the treatments T8 and T7 were found to have a significant increase with a value of 55.05 ± 15.7% and 54.08 ± 9.64%.

**Figure 6:**
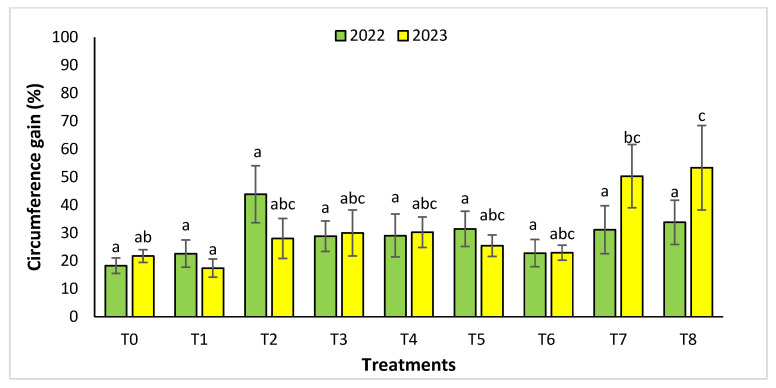
Circumference gain (%) for the Rasmouka site in two years (2022–2023). The bars with the same letters are not significantly different at a 5% significance level, according to the Tukey test. Error bars refer to standard errors.

For the circumference parameter (Figure 6), the treatments that increased significantly this parameter were T8 and T7 with a value of 53.36 ± 15.11% and 50.34 ± 11.29% in 2023, contrary to the year 2022. during which we did not find any significant difference.

### 2.2. Physicochemical Parameters of Leaves

**Figure 7:**
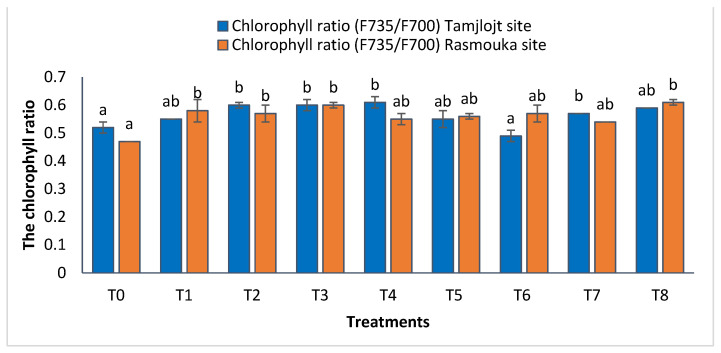
Chlorophyll ratio and the antioxidant activity of Tamjlojt and Rasmouka sites. The bars with the same letters are not significantly different at a 5% significance level, according to the Tukey test. Error bars refer to standard errors.

For the Tamjlojt site, the chlorophyll ratio F735/F700 results revealed significant increases in this parameter for the different treatments (Figure 7), with the most improved treatments being T4, T3, T2, T8, and T7 with values of 0.61 ± 0.02, 0.60 ± 0.02, 0.60 ± 0.01, 0.59 ± 0.00 and 0.57 ± 0.00, respectively. The change in the ratio of F735/F700 can reflect the strength of leaf photosynthetic activity and the amount of light energy used by plants.

**Figure 8:**
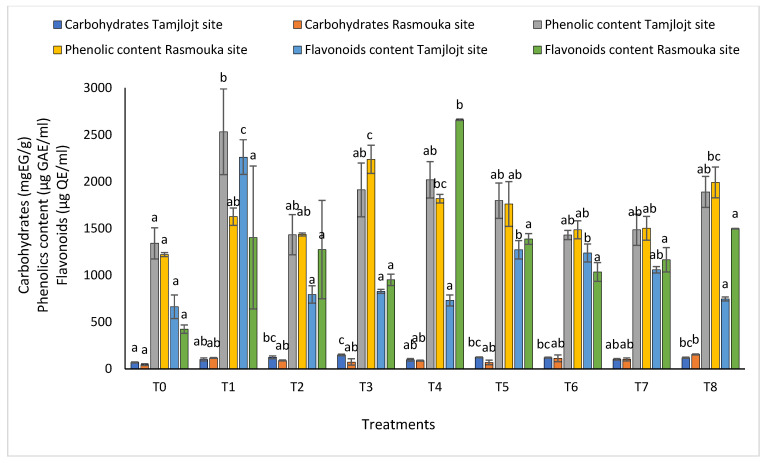
The carbohydrate, phenolic, and flavonoid content of Tamjlojt and Rasmouka sites. The bars with the same letters are not significantly different at a 5% significance level, according to the Tukey test. Error bars refer to standard errors.

For carbohydrates (Figure 8), the different treatments significantly improved this parameter, the most important being are T3, T5, T2, T6, and T8 with the following values: 148.89 ± 8.11, 122.76 ± 15.37, 123.12 ± 4.72, 122.76 ± 15.37, 119.27 ± 5.60, and 118.40 ± 6.89 (mg EG/g), respectively.

**Figure 9:**
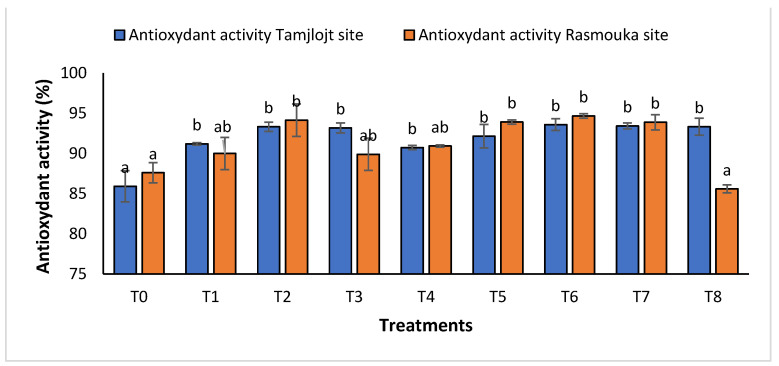
The antioxidant activity of Tamjlojt and Rasmouka sites. The bars with the same letters are not significantly different at a 5% significance level, according to the Tukey test. Error bars refer to standard errors.

For the concentration of phenolic, all treatments showed a significant increase in the concentration of this compound, and the treatment with the highest concentration was T1 with a value of 2532 ± 457.13 (µg GAE/mL). For flavonoids, the treatments that showed a significant effect were T1, T6, and T5 with the following values: 2261.98 ± 184.61, 1237.70 ± 95.65 and 1271.85 ± 99.08 (µg QE/mL). For DPPH activity (Figure 9), all treatments showed a significant difference for this parameter within a range of (93.60 ± 0.73% to 91.20 ± 0.15%).

For the Rasmouka site, the results of the chlorophyll ratio F735/F700 showed that the different treatments significantly improved this parameter; the treatments that improved the most were T8 and T3 with values of 0.60 ± 0.01 and 0.61 ± 0.01, respectively (Figure 7). For carbohydrates, the different treatments significantly improved this parameter; the most important ones are T8, T1, T7, and T6, with values of 153.81 ± 4.59, 116.57 ± 4.22, 101.32 ± 16.05 and 112.24 ± 35.97 (mg EG/g), respectively.

For the concentration of phenolic, all treatments showed a significant increase in the concentration of this compound; the treatment with the highest concentration was T3 with a value of 2237.43 ± 150.20 (µg GAE/mL), followed by T8 with a value of 1991.81 ± 164.36 (µg GAE/mL). The T6 and T2 treatments have the lowest concentration, with values of 1485.38 ± 96.10 and 1435.53 ± 15.95 (µg GAE/mL), respectively (Figure 8). For flavonoids, the treatment that showed a significant effect was T4 with 2660.74 ± 8.55 (µg QE/mL).

For DPPH activity (Figure 9), all treatments showed a significant difference for this parameter in the range of (89.99 ± 1.99% to 94.66± 0.27%), except for the T8 treatment with a value of 85.59 ± 0.15%.

### 2.3. Physicochemical Parameters of Soil

**Figure 10:**
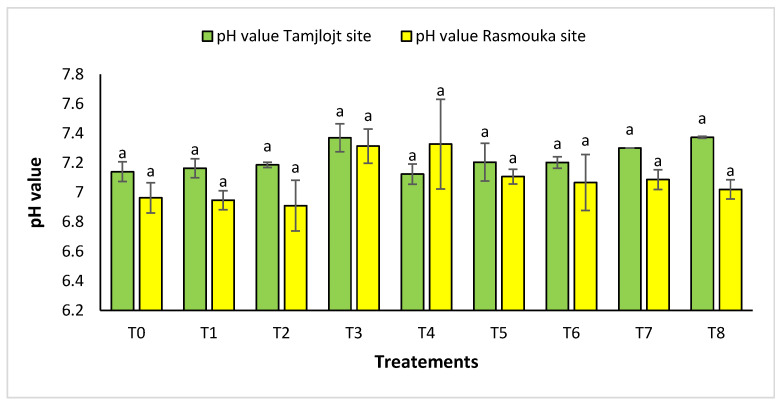
pH value of Tamjlojt and Rasmouka sites. The bars with the same letters are not significantly different at a 5% significance level, according to the Tukey test. Error bars refer to standard errors.

**Figure 11:**
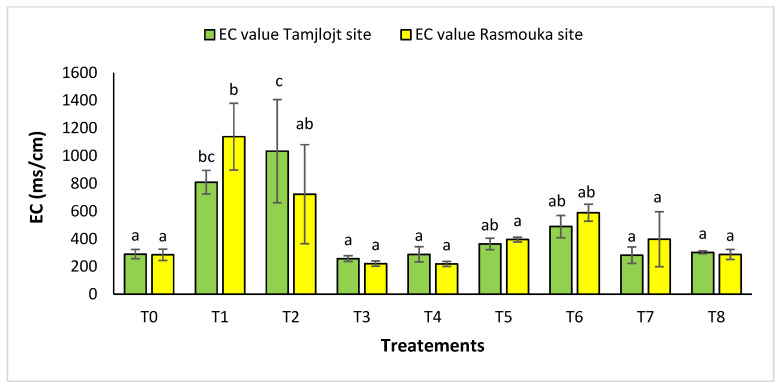
Electrical conductivity of Tamjlojt and Rasmouka sites. The bars with the same letters are not significantly different at a 5% significance level, according to the Tukey test. Error bars refer to standard errors.

For pH, there was no significant difference between the two sites for the different treatments (Figure 10). For the Tamjlojt site, the pH value was between 7.12 ± 0.06 and 7.37 ± 0.09; for the Rasmouka site, the values were between 6.91 ± 0.17 and 7.32 ± 0.30. For electrical conductivity at the Tamjlojt site (Figure 11), the treatments that increased this parameter significantly were T2 and T1 with values of 1034.67 ± 372.94 and 810.76 ± 84.85 ms/cm; for the Rasmouka site, treatment T1 increased this parameter significantly to 1139.00 ± 241.30 ms/cm.

**Figure 12:**
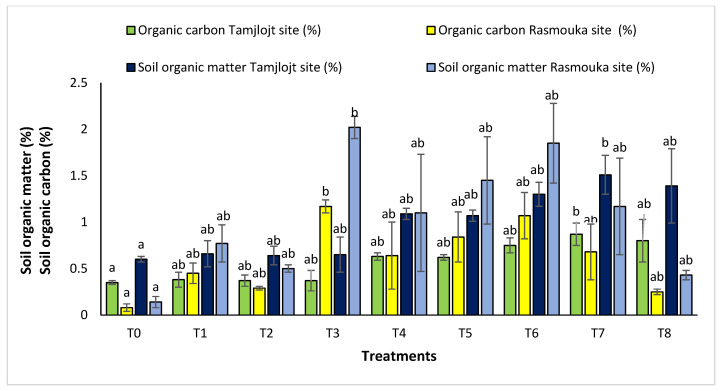
Organic matter soil and organic carbon of Tamjlojt and Rasmouka. The bars with the same letters are not significantly different at a 5% significance level, according to the Tukey test. Error bars refer to standard errors.

For organic carbon, all the treatments increased the percentage of this parameter in the soil. For the Tamjlojt site, the T7 treatment significantly improved this parameter with a value of 0.87 ± 0.12%, as well as the organic matter content, which improved by a value of 1.51 ± 0.21% (Figure 12). For the Rasmouka site, the T3 treatment increased the percentage of organic carbon with a value of 1.17 ± 0.07%, and the organic matter content also improved with a value of 2.02 ± 0.12%.

**Figure 13:**
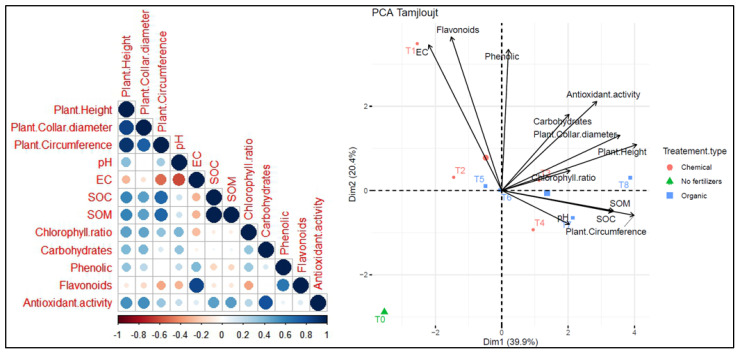
Principal component analysis (PCA) illustrates the variations between the studied parameters and the different treatments used for the growth of argan plants in the Tamjlojt site. pH: potential of hydrogen, EC: electrical conductivity, SOM: soil organic matter, SOC: soil organic carbon.

Two principal component analyses (PCA) were conducted to assess the relationship between different treatments and parameters across two distinct sites. At the Tamjlojt site (Figure 13), PC1 and PC2 accounted for 39.9% and 20.4% of the data variability, respectively. The agronomic parameters (plants height, collar diameter, circumference) show a positive correlation with chlorophyll ratio, carbohydrates, phenolic and antioxidant activity. Soil pH is positively correlated with variables such as SOC and SOM. Moreover, it shows a negative correlation with electrical conductivity and flavonoids. The agronomic parameters and leaf parameters (carbohydrates, phenolic and antioxidant activity) were positively correlated with T8 and T3 as organic fertilizers. Treatment T7 is associated with pH and soil organic carbon (SOC), and T1, T2 and T5 were positively correlated with electrical activity and flavonoids. The T0 treatment without fertilizers shows distinct characteristics compared to the other treatments.

**Figure 14:**
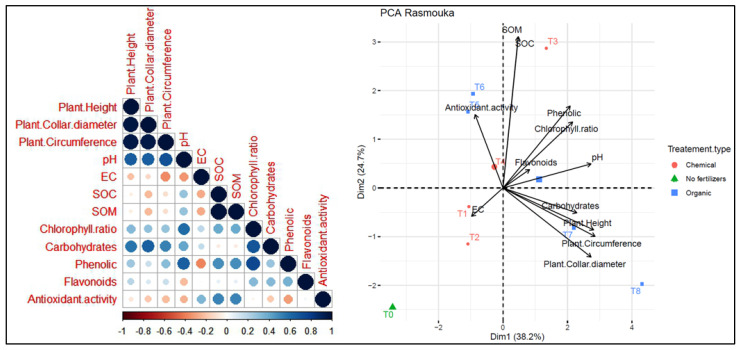
Principal component analysis (PCA) illustrates the variations between the studied parameters and the different treatments used for the growth of argan plants in the Rasmouka site. pH: potential of hydrogen, EC: electrical conductivity, SOM: soil organic matter, SOC: soil organic carbon.

On the other hand, at the Rasmouka site (Figure 14), PC1 and PC2 explained 38.2% and 24.7% of the data variability, respectively. Agronomic parameters and leaf parameters were positively correlated with treatments T8, T7, T4, and T3. However, they were negatively correlated with antioxidant activity. Plants height shows a positive correlation with collar diameter, circumference and carbohydrates. Soil pH shows a positive correlation with variables such as chlorophyll ratio, phenolic, carbohydrates, SOC and SOM. Moreover, it shows a negative correlation with antioxidant activity. Electrical conductivity (EC) has a negative correlation with pH, chlorophyll ratio, phenolics and flavonoids. The agronomic parameters and carbohydrates were positively correlated with T8 and T7 as organic fertilizers. Treatment T3 is associated with soil organic carbon (SOC), and T1 and T2 were positively correlated with electrical activity, while T5 and T4 were positively correlated with antioxidant activity. The T0 treatment without fertilizers shows distinct characteristics compared to the other treatments.

## 3. Discussion

### 3.1. Vegetative Growth Parameters

Considering the vegetative growth parameters, the application of inorganic and organic fertilizers significantly improved the height, collar diameter, and growth of *Argania Spinosa*. These findings align with those of Haberman et al. [29], who reported that the mean relative increase in trunk circumference was lowest at the N0 level (64%) and progressively higher for trees receiving higher nitrogen levels, ultimately achieving a mean of 77% at the N300 level. Mazeh et al. [30] reported that both chemical and organic fertilization on Young Potted Olive Trees significantly increased the growth of the trees with respect to the control, which is similar to our results. The organic fertilizer determined a higher growth than the chemical one, showing a higher increment in both collar diameter (+22%) and tree height (+30%). The organic fertilizer has also been reported to have a biostimulant action due to the high content of amino acids and protein, similar to the results found by Erel et al. [31], which show that the collar diameter of olive seedlings is significantly influenced by nitrogen and phosphorus concentrations; the treatments deficient in nitrogen and phosphorus caused smaller collar diameters, while potassium had no effect. Also, Jakhro et al. [32] found that the plant height and collar diameter of *Olea europeae* responded significantly to the increased NPK (50:25:25) and FYM (2:2), followed by treatment P (25 g alone), treatment N (50 g alone), and treatment K (25 g alone).

The importance of phosphorus was confirmed by Jiménez-Moreno et al. [33], who showed that vegetative growth showed a reduction in growth at the lower doses of P application. However, [34] showed that tree growth (annual shoot elongation) was positively affected by fertilization, especially by nitrogen fertilization. However, collar diameter growth did not respond to phosphorus or nitrogen soil fertilization.

### 3.2. Physicochemical Parameters of Leaves

The results showed that the application of chemical and organic fertilizers significantly increased the photosynthetic activity, which is represented in chlorophyll ratio. In the Rasmouka site, all the treatments showed a significant increase in this parameter. In fact, several studies demonstrated a direct association between fertilization with some minerals and chlorophyll content, which plays a crucial role in plant photosynthesis. For example, Zivdar et al. [35] showed that foliar application of potassium significantly increased the leaf chlorophyll content of all *Olea europeae* cultivars compared to control treatments. Additionally, Roca et al. [36] found that the application of N significantly increased both the plant height and leaf chlorophyll content in young olive trees, with high N treatment in young *Olea europeae* plants. Furthermore, it was found that chlorophyll content is related to leaf nitrogen content due to the underlying incorporation of nitrogen into chlorophyll molecules [37,38].

The carbohydrate, phenolic and flavonoid contents were also significantly influenced by the different treatments compared to the control. Our results showed a positive effect of compost and NPK fertilizers on carbohydrates, phenols, and flavonoids on *Argania spinosa* leaf, which is confirmed by Sarwar et al. [39], who found that the application of compost and NPK fertilizer had a positive effect on improving the biochemical and antioxidant properties of *Moringa oleifera* leaf over the control. These findings are in accordance with Gendy et al. [40], who reported that the application of bio-fertilizer alone or with a combination of nitrogenous fertilizers increased carbohydrate and flavonoid contents in the plant species. Similarly, a study conducted by Muscolo et al. [41] showed that organic fertilizers enhanced the synthesis of total phenols, flavonoids, and anthocyanins, along with the antioxidant activities of red Topepo sweet peppers compared to those grown in unfertilized soil. In addition, Ibrahim et al. [42] showed that total phenolics and flavonoids were influenced by fertilizer source and fertilization rates. It was observed that the application of organic fertilizer increased the production of total phenolic and flavonoid contents in *L. pumila Benth*. 12% and 22%, respectively, in the organic fertilizer treatment compared to inorganic fertilization. However, other studies that found lower levels of total phenols and phenolic compounds in plants grown with organic amendments (compost) compared to those grown in control soil of Rosmarinus officinalis plants [43].

### 3.3. Physicochemical Parameters of Soil

Concerning soil proprieties, pH and EC indicate good nutrient availability for plants. Soil pH value was slightly increased in the soils that were treated with organic and inorganic fertilization but without any significant difference. Many studies revealed the effect of fertilization on EC such as that Hati et al. 2007, which reported that electrical conductivity values of (EC) increased by application of 100% NPK + FYM and 100% NPK treatments, being significantly higher than in control and 100% N treatments. In the 100% NPK + FYM and 100% NPK treatments, the EC was 0.13 and 0.10 dS m^−1^, respectively, higher than the initial value. Composted plant residues and the increment attributed to the release of soluble salts of composted materials during their decomposition were studied in [44], and Rajesh Kishor Tripathi found that a maximum increase in Ec was seen under the combined application of NPK and a minimum under no fertilizer. Similarly, [45] indicates an increasing trend in Ec level with phosphate application under different cropping system

For organic carbon, all treatments significantly increased the percentage of this parameter in the soil. These results are in accordance with those reported by Liu et al. [46], who showed that fertilization significantly increased the SOC content or stock of agricultural soils, with an average effect size of 0.2707 ± 0.0086, and with [47], which found that compared with those under no fertilization, all fertilization treatments (organic and inorganic) significantly increased soil SOM content of rice soil, similarly to so many studies [48,49,50] that showed that the application of NPK to soil increased the organic matter content. Their research demonstrates a direct correlation between the application of these nutrients and the improvement in soil quality. The results showed that the highest organic carbon content was recorded on the site that received the combined NPK application, closely followed by NP. Multinutrient combined fertilization (NP, NK, PK, NPK) has a greater impact on soil organic carbon (SOC) than single-nutrient fertilization (N, P, K) due to its ability to provide balanced nutrition for plant and microbial populations [51].

## 4. Materials and Methods

### 4.1. Study Site

This experiment was carried out in the arganiculture platforms among the agricultural development project in vulnerable areas (DARED), which aims to establish argan orchards on 10,000 hectares in the first phase within the framework of the improvement in the technical management of arganiculture in orchards and monitoring of agronomic and physiological comportment of argan plantations.

**Figure 15:**
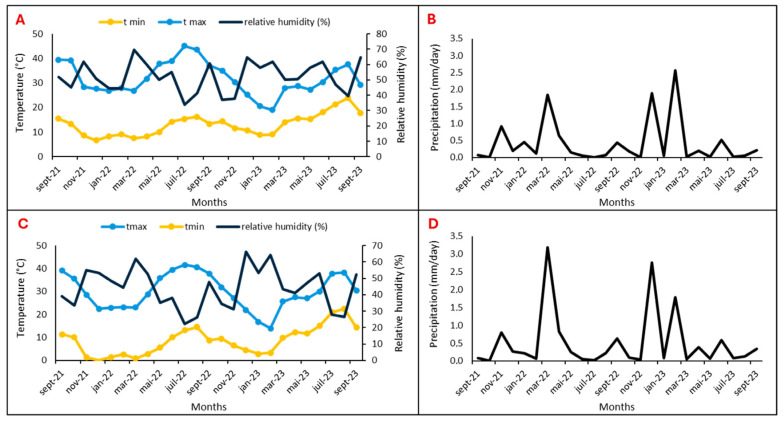
Temperature, relative humidity, and precipitation of Tamjlojt (**A**,**B**) and Rasmouka sites (**C**,**D**).

Two argan fields located in the Souss Massa vulnerable areas, represented by two sites, Tamjlojt (29°57′53.34″ N, 9°21′23.62″ W) and Rasmouka (29°45′17.47″ N, 9°32′1.96″ W), were selected for organic and mineral fertilization tests. Therefore, the present study was conducted during two consecutive study seasons (2022 and 2023) on young argane trees (four years old) planted at 4 × 10 m and irrigated twice a month (Table 1). The temperatures, relative humidity and precipitation for the two sites are illustrated in Figure 15.

### 4.2. Field Experiments and Treatments

To evaluate the effect of different doses of compost and NPK fertilizers on the growth characteristics, soil proprieties, biochemical and antioxidant potential of *Argania spinosa*, eight treatments were applied (T1, T2, T3, T4, T5, T6, T7, and T8). T0 was a control without fertilizer application (Table 2).

**Figure 16:**
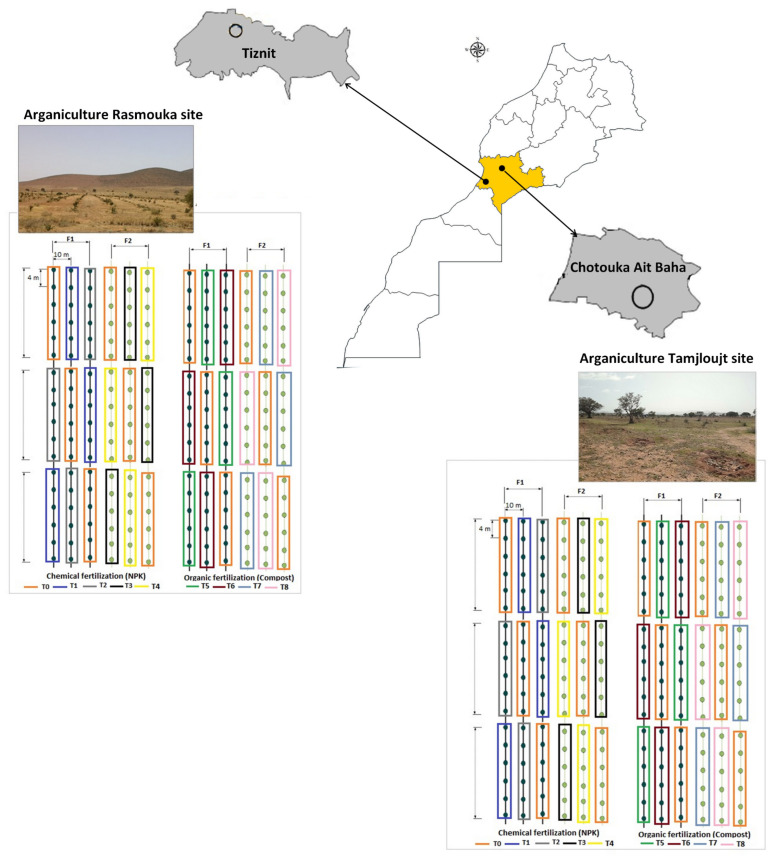
Schematic diagram of the distribution of the experimental design (T: treatments, F1: two fertilizers’ application per year and F2: once per year).

The characteristics of compost used are presented in Table 3. Four experiments were carried out at each site. The application of different treatments in each experiment was performed using a completely randomized design with three replicates. Each replicate consisted of six plants (Figure 16). The fertilizers were applied twice in season F1 (Mars and September) and once in season F2 (Mars) each year.

### 4.3. Vegetative Growth Parameters

The growth parameters, plant height, collar diameter, and circumference rate, were assessed three times per year. Plant height was measured on 18 selected plants for each treatment using a meter ruler. The collar diameter was measured at a marked point, 3 cm above the ground level, using slide calipers. These parameters were taken and transformed to percentages to express the relative increase.

### 4.4. Physicochemical Parameters of Leaves

For chlorophyll content, 30 leaves from each treatment were used to determine the ratio F735/F700, which indicates chlorophyll levels using a chlorophyll content meter (ccm-300). A mixture of fresh leaves samples from the 18 plants selected for each treatment to constitute the three replicates was harvested at the end of experiment and dried in the shade followed by oven drying at 45 °C for 72 h.

#### 4.4.1. Carbohydrates’ Analysis

The phenol-sulfuric acid method [52] was used to determine the carbohydrate content. 20 mg of dried and crushed leaves were mixed with 2 mL of ethanol (70%). The mixture was then centrifuged twice at 2000× *g* for 10 min, and the supernatant was added to 16 mL of demineralized water. Subsequently, 200 µL of the diluted extract was added to 200 µL of phenol (5%) and 1 mL of concentrated sulfuric acid. The absorbance was observed at 490 nm using a spectrophotometer visible (ONDA V-10 plus, Shanghai, China) against the reagent blank [53]. The results were expressed as mg g^−1^ of standard glucose equivalents.

#### 4.4.2. Determination of Phenolic Content

The determination of total phenolic compounds was performed based on the spectrophotometric method using the Folin–Ciocalteu reagent [54]. An aliquot of 400 µL of each methanolic extract (20 mg/mL) was added to 2000 µL of diluted Folin–Ciocalteu reagent (10%) and 1600 µL Na_2_CO_3_ solution (7.5%). The solutions were incubated in the dark at room temperature for 30 min following the absorbance reading at 765 nm using a spectrophotometer (ONDA V-10 plus) and compared to a gallic acid calibration curve. The results were expressed as µg of gallic acid equivalents per mL of stock solution (GAE/mL).

#### 4.4.3. Determination of Total Flavonoids

The concentration of total flavonoids was determined based on the method of a flavonoid–aluminum formation [55]. Briefly, an aliquot of 2 mL of the methanolic extract was added to 2 mL of AlCl_3_ (2%). After incubation for 30 min, the absorbance was measured at 430 nm using a spectrophotometer (ONDA V-10 plus, P.R.C).

The Catechin was used to establish the standard curve, and the results were expressed as µg of quercetin equivalent per mL of stock solution (QE/mL).

#### 4.4.4. Determination of DPPH Radical Scavenging Activity (%)

The antioxidant activity of samples was determined by the determination of the radical scavenging activity using the 2,2-diphenyl-1-picrylhydrazyl (DPPH) method as described in [56]. Then, 100 µL of ethanolic extract was added to 3900 µL of DPPH prepared with methanol (0.025 g/L) [57]. After 20 min of incubation in the dark at room temperature, the absorbance was measured at 515 nm using a spectrophotometer (ONDA V-10 plus, P.R.C).

The antioxidant activity percentage was calculated using the following formula:

Antioxidant activity (%) = [(Ac − As) ÷ Ac] × 100

Ac: control reaction absorbance (DPPH); As: testing sample absorbance.

### 4.5. Physicochemical Parameters of Soil

Soil samples were collected from the experimental site at the 0–30 cm soil layers using an auger, and a mixture of soil samples from the 18 plants selected for each treatment to constitute the three replicates was sampled at the end experiment and used for the analysis of physicochemical properties such as soil pH, organic carbon (OC), total N, available P, and exchangeable bases (Ca, Mg, and K), which were analyzed after treatments. The samples were dried, stored at room temperature in the laboratory, and then analyzed.

Then, 25 g of air-dried soil was dissolved in 50 mL of distilled water. After that, a mechanical shake at 15 rpm for 1 h was carried out to dissolve soluble salts. After standardization, soil pH was measured using a pH meter (Consort™ C3010, Angers, France), and electrical conductivity (EC) was determined with an EC meter (Consort™ C3010). The total N content of the soil was determined by the procedure of the Kjeldahl method [58]. Available P was determined using the Olsen method [59]. For K^+^, Na^+^, and Ca^2+^ exchangeable basic cations were determined using a flame photometer (BWB XP). To determine soil organic matter, the Walkley–Black method was used [60].

### 4.6. Statistical Analysis

Data analysis was subjected to ANOVA using the R Studio-2024.04.1-748 program (Posit Software, Boston, MA, USA, PBC. 2024). Data for the growth of *A. spinosa* seedlings are presented as means with error standards. Any difference mentioned is significant at *p* < 0.05 using the Tukey test.

## 5. Conclusions

This work represents the first fertilizer application to the *A. spinosa* domestication program, a species naturally adapted to semi-arid conditions. It is essential to optimize its growth and increase its leaf biochemical properties (chlorophyll, carbohydrates, and secondary metabolites). By providing nutrients such as nitrogen, phosphorus, and potassium in balanced quantities, we can increase the tree’s resistance to environmental challenges while maximizing its agricultural potential. This approach not only supports sustainable cultivation practices but also preserves the environmental balance of the *A. spinosa* habitats, safeguarding the long-term health and productivity of this precious species. As there are no previous studies, it is concluded from this study that mineral and organic fertilization has a positive effect on improving the growth characteristics, soil proprieties, biochemical and antioxidant potential of *A. spinosa*. Based on our present results it can be concluded that it is recommended to fertilize argan plants with T4 and T3 as a chemical fertilizer in the Tamjlojt site, and with T7 and T8 as an organic fertilizer in the Rasmouka site to obtain the maximum vegetative growth. Understanding the specific nutrient requirements of *A. spinosa* is crucial for sustainable cultivation practices, ensuring its resilience and long-term viability. For future studies, it might be interesting to conduct similar research on the different doses of fertilizers according to the phenological periods of *A. spinosa*.

## Figures and Tables

**Table 1:** Localization and soil characteristics of the experimental sites.

Experimental Site	Localization	Soil Characteristics							
		pH	EC (ms/cm)	SOM%	P mg/kg	Na^+^ ppm	Mg^2+^ mg/kg	Ca^2+^ mg/kg	K^+^ mg/kg
Rasmouka/ (Tiznit)	29°45′17.47″ N 9°32′1.96″ W	7.59	250	3	12.5	8.2	180	200	90
Tamjlojt/(Chtouka Ait Baha)	29°57′53.34″ N 9°21′23.62″ W	7.99	200	1.1	14	8.2	235	300	100

pH: potential of hydrogen, EC: electrical conductivity, SOM: soil organic matter, P: phosphorus, Na^+^: sodium ion, Mg^2+^: magnesium ion, Ca^2+^: calcium ion, K^+^: potassium, ppm: parts per million, N: north, W: west.

**Table 2:** The different doses of compost and NPK fertilizers applied.

Treatments	Fertilizers	Dose			Frequency/Season
		N	P	K	-
T0	Control	0	0	0	-
T1	Chemical fertilizers g/plant	80	50	70	F1
T2		125	75	100	F1
T3		160	100	140	F2
T4		250	150	200	F2
T5	Organic fertilizers Kg/plant	2.5			F1
T6		5			F1
T7		5			F2
T8		10			F2

**Table 3:** Physical and chemical properties of the experimental compost.

Characteristics	pH	EC (ms/cm)	SOM%	COT (%)	P mg/g	N (%)	Humidity (%)
Compost	7.31	12.97	56.64	31.38	5.653	0.84	42.67

## Data Availability

All data generated in this work are provided within this manuscript.
